# Changes in muscle activation with graded surfaces during canter in Thoroughbred horses on a treadmill

**DOI:** 10.1371/journal.pone.0305622

**Published:** 2024-06-14

**Authors:** Yuji Takahashi, Toshiyuki Takahashi, Kazutaka Mukai, Yusaku Ebisuda, Hajime Ohmura

**Affiliations:** Sports Science Division, Division of Equine Research Institute, Japan Racing Association, Shimotsuke, Tochigi, Japan; Loughborough University, UNITED KINGDOM

## Abstract

Understanding how muscle activity changes with different surface grades during canter is essential for developing training protocols in Thoroughbreds because canter is their primary gait in training and races. We measured the spatiotemporal parameters and the activation of 12 surface muscles in the leading limb side of 7 Thoroughbreds. Horses were equipped with hoof strain gauges and cantered at 10 m/s on a treadmill set to grades of −4%, 0%, 4%, and 8%, randomly, for 30 seconds each without a lead change. Integrated electromyography (iEMG) values during stance and swing phases were calculated and normalized to mean iEMG values during stride duration at 0% grade in each muscle. The iEMG values at each grade were compared using a generalized mixed model. Stride duration significantly decreased due to shorter swing duration on an 8% grade (P < 0.001) compared to all other grades, where no significant changes were observed. Compared to a 0% grade, the normalized iEMG values during the stance phase on an 8% grade in five muscles significantly increased (*Musculus infraspinatus*; +9%, *M*. *longissimus dorsi* (LD); +4%, *M*. *gluteus medius* (GM); +29%, *M*. *biceps femoris*; +47%, *M*. *flexor digitorum lateralis*; +16%). During the swing phase, the normalized iEMG values in six muscles significantly increased on an 8% grade compared to a 0% grade (*M*. *splenius*; +21%, *M*. *triceps brachii*; +54%, LD; +37%, GM; +24%, *M*. *semitendinosus*; +51%, *M*. *extensor digitorum longus*; +10%). No significant changes were observed in iEMG values on −4% and 4% grades compared to the 0% grade. Although +/− 4% grades had little effect on neuromuscular responses, 8% uphill canter reduced stride duration due to decreased swing duration and required increase of muscle activation during either stance and swing phase. Canter on an 8% grade might strengthen equine muscles to increase propulsive force and stride frequency.

## Introduction

Neuromuscular adaptations in response to environmental changes are essential for athletic horses competing in horse racing or cross-country events because they often run on courses with uphill and downhill sections at considerably high speeds [1–4]. Locomotory muscles play an important role in raising the horses’ center of mass against gravity when running uphill or in absorbing the loss of potential energy during downhill running [5–12]. Thus, a better understanding of the effects of gradients on the functional activity of locomotor muscles in racehorses is key to developing effective training and rehabilitation programs to maximize racing performance and prevent overload injuries in the skeletal muscles.

Recent studies have explored how stride parameters and muscle activity change on inclined or declined treadmills at a trotting gait using electromyography (EMG) [9, 13–16]. It has been observed that during trotting at a given speed, the stride duration on graded surfaces did not change [9, 13, 14, 16] or slightly increased with incline [15, 17] when compared to level surfaces. The activity of locomotor muscles, such as the *Musculus vastus lateralis*, lateral head of *M*. *triceps brachii* (TB), *M*. *gluteus medius* (GM), *M*. *biceps femoris* (BF), *and M*. *longissimus dorsi* (LD) generally increases on uphill gradients [9, 13–16]. However, the activity of *M*. *extensor digitorum longus* (EDL) and *M*. *gastrocnemius lateralis* did not significantly change with incline [9]. In contrast, only the activity of GM decreased on downhill gradients, with no changes in stride duration [9]. These results indicate that different muscles show different neuromuscular responses to graded running, according to their specific functions. Further, a trotting horse on an incline increases the required power by increasing work per cycle, that is, they increase muscle activation without increasing the number of cycles [15].

Understanding muscle behavior during canter is important for understanding the locomotion of quadrupeds and improving training and rehabilitation programs for Thoroughbred racehorses, because canter is the gait unique to quadrupeds and the primary gait for Thoroughbreds during high-intensity exercises. With regard to stride parameters during uphill canter, studies have reported either no change [18] or a decrease in stride duration because of a decrease in swing duration [19, 20]. Although changes in stride parameters during canter may differ from those observed in a trot, it is hypothesized that muscle activity increases during uphill canter, because oxygen consumption increases on uphill grades, regardless of the gait [21, 22]. Due to increased mass-specific peak vertical force in the hindlimb [19], hindlimb muscle activity would increase to elevate the center of mass during the stance phase. However, to date, only one study has shown increases in muscle activity on an uphill treadmill during canter, focusing on forelimb muscles such as the *M*. *brachiocephalicus* (Br) and TB, which could facilitate faster limb protraction in a short swing duration [20]. Conversely, downhill exercise requires less oxygen consumption during canter compared to a level surface [23], suggesting fewer muscle fiber recruitments, because eccentric contraction occurs during the stance phase [24]. However, no study has investigated the effects of downhill gradients on stride parameters and muscle activity during canter. Investigating the impacts of graded surfaces on muscle activity during canter could offer novel insights.

Although both uphill and downhill canter would change muscle activity patterns, the effects of incline on muscle activity would not be uniform for all muscles because of the different roles of different muscles [25, 26]. The present study aimed to characterize muscle activity patterns that occur in horses cantering on graded surfaces, focusing on their functions. For this purpose, we examined the activity of 12 surface muscles as horses cantered on a treadmill at different gradients. Specifically, we hypothesized that muscle activity in the hindlimbs during the stance phase would increase during uphill canter and decrease during downhill canter when compared to cantering on a level surface. In addition, muscle activity in both forelimbs and hindlimbs during the swing phase would increase in uphill canter because uphill canter required faster limb protraction in a shorter swing duration [19], whereas no significant changes in muscle activity during the swing phase are expected downhill canter, based on observations of no changes in swing durations in downhill walking and trotting [9, 16].

## Materials and methods

### Ethics

The study protocol was approved by the Animal Care and Use Committee of the Equine Research Institute of Japan Racing Association (approval no. 20–7).

### Horses

We used seven clinically healthy Thoroughbreds (three geldings and four mares) for the study. The median age of the horses was 4 (range, 3–8) years, and their average body weight, represented as mean ± standard deviation, was 528 ± 62 kg. All horses were familiar with running on a treadmill. Before the experiments, they underwent training sessions, cantering at 10 m/s under four different grades (−4%, 0%, 4%, and 8%) on a treadmill (SÄTO AB, Knivsta, Sweden). This training, conducted twice a week for three weeks, was aimed at habituating the horses to each grade. In addition, the preferred canter lead of each horse was determined and recorded during training sessions.

### Experimental setup

The surface EMG (sEMG) signals from 12 muscles: *Musculus splenius* (SP), *M*. *brachiocephalicus* (Br), *M*. *infraspinatus* (Inf), long head of *M*. *triceps brachii* (TB), *M*. *common digitorum extensor* (CDE), *M*. *longissimus dorsi* (LD), *M*. *tensor fasciae latae* (TFL), *M*. *gluteus medius* (GM), *M*. *biceps femoris* (BF), *M*. *semitendinosus* (ST), *M*. *extensor digitorum longus* (EDL), and *M*. *flexor digitorum lateralis* (FDL) were recorded. As per previous literature [13, 27–30], silver–silver chloride electrodes (H124SG, Covidien, MA, USA), each 16 mm in diameter, were attached to each muscle, parallel to the muscle fibers. The electrodes were spaced 25 mm apart, center-to-center, with active and reference electrodes placed next to each other. To ensure that the targeted muscle at each electrode location was not covered by other muscles, we used ultrasonography as well as the results of dissections from horses euthanized for other research projects. Detailed descriptions of electrode positions for each muscle are available in the S1 Appendix.

Before electrode attachment, the skin over and around each muscle belly was shaved and cleaned with alcohol. The electrodes were then attached to the muscles on the lead side during the canter using a fast-acting glue (Gachi; Kokuyo Co., Osaka, Japan). The sEMG data were collected in the same way as in our previous studies [28–30]. Briefly, the electrodes were connected to a compact electrode telemetry system, equipped with active, reference, and ground electrodes, including a wireless transmitter (ZB-150H; Nihon Kohden Corp., Tokyo, Japan), via snap-type lead cables (TK-217-018; Unique Medical Co., Tokyo, Japan). The sEMG data from the transmitters, attached to the horses’ bodies via a foam pad (Foam pad 75A; Nihon Kohden Cop., Tokyo, Japan), were stored and displayed in real time on a host computer (WEB-7000; Nihon Kohden Corp., Tokyo, Japan).

To detect foot-on and foot-off events, strain gauges (N22-FA-10-120-11-VS3; Showa Measuring Instruments, Inc., Tokyo, Japan) were firmly attached to the dorsal midline of the fore and hind hooves of the lead side using glue (Gachi; Kokuyo Co., Osaka, Japan). Moreover, the sEMG and strain gauge data were synchronized by connecting dynamic strain-measuring instruments (DPM-612B; Kyowa Electronic Instruments Co., Tokyo, Japan) to the input terminal box (JC-130H; Nihon Kohden Corp., Tokyo, Japan) of the multichannel telemetry system using a coaxial cable with Bayonet Neill–Concelman connectors. Both sEMG and hoof-strain gauge signals were collected at a frequency of 1000 Hz, with a 10-bit resolution (5 mV, full scale). The signals were then filtered using a band-pass filter (including a Bessel filter ranging 30–500 Hz for EMG) and a low-pass filter set at 250 Hz for the strain gauges. Data showing high baseline levels even after applying this filter or those in which detachment of electrodes occurred during measurement were omitted from the analysis.

### Experimental protocol

The experiment was conducted on a treadmill. Each horse began with a warm-up consisting of a 1-minute walk at 1.7 m/s, followed by a 3-minute trot at 3.5 m/s on a flat treadmill. Subsequently, the horses cantered at 10 m/s for 30 seconds under four different grades (−4%, 0%, 4%, and 8%), in a randomized order. Before cantering at each grade, the horses trotted on the same grade for 45 seconds. Trials were conducted on the horses preferred canter lead. If the horse changed canter lead during the trial, the speed was reduced to help transition the horse back to a trot; then the horse was encouraged to canter again on the preferred canter lead.

### Data analysis

Based on previous literature [31, 32], the foot-on and foot-off events were defined as the moments when strain values started to increase and returned to their initial levels, respectively. Stride duration was defined as the interval between two hindlimb foot-on events. Stance duration was defined as the time between foot-on and the subsequent foot-off event, and swing duration as the time between a foot-off event and the next foot-on event for an individual leg. After the removal of DC offset and full-wave rectification of the sEMG signal, the iEMG value, which represents the area under the voltage curve of the EMG waveform, was calculated during the stance phase and swing phase. Forelimb stance and swing duration were used to calculate iEMG values in SP, Br, Inf, TB, and CDE, while hindlimb stance and swing duration were used in LD, GM, TFL, BF, ST, EDL, and FDL. The iEMG values of each muscle from each stride were normalized against the associated mean iEMG values of each horse, calculated over 10 consecutive strides during a 0% grade. This approach aligns with a study investigating muscle activity changes in healthy young humans running on graded surfaces [33]. Furthermore, muscle activation onset and offset were detected using enveloped signals, which were smoothed using a fourth-order low-pass Butterworth filter with a 10-Hz cutoff frequency. This method aligns with a recent publication [34] and was implemented via a custom-made MATLAB script (Release 2022b; The MathWorks Inc., Natick, MA, USA). The amplitude threshold for detecting muscle activation was set at 10% of the peak amplitude for each sEMG signal, and the timing threshold was defined as 5% of the average gait cycle duration across all horses. The onset and offset events for each muscle were normalized to a 0%–100% scale of the stride cycle starting at the contact of each hoof.

For statistical analysis, we used the mean data from 10 consecutive strides. All statistical analyses were performed using commercial software (SAS version 9.4; SAS Institute, Cary, NC, USA). To evaluate the effects of grade on stride parameters, including stride duration, stance duration, swing duration, and duty factor (defined as the proportion of a stride in which the hoof is in contact with the ground), as well as normalized iEMG values during the stance and swing phases, and the timing of muscle onset or offset, we used a generalized linear mixed model with a gamma distribution and log link function (PROC GLIMMIX). In this model, the grade condition was treated as a fixed effect, while each horse was considered as a random effect. In addition, we employed Tukey’s multiple comparisons of least-squared means to characterize the differences between grades. A P value of less than 0.05 was considered statistically significant.

## Results

In our study, three horses cantered on the left lead, whereas four horses cantered on the right lead. The strain gauge disconnected in two horses at different time points during data collection, which led to the exclusion of associated sEMG data (forelimb muscles for one horse on all grades and hindlimb muscles for one horse on the 8% grade condition) given the inability to detect stride cycles. In addition, sEMG data from Br, TB, CDE, and EDL were excluded across 4 horses (see S1 Table) and thus not included in the statistical analysis. All data used for the analysis is available in S1 Table.

We found that grade significantly affected stride duration (P < 0.001), forelimb stance duration (P = 0.03), forelimb swing duration (P = 0.002), hindlimb swing duration (P < 0.001), and the duty factor of the hindlimb (P = 0.008, Table 1). However, the stance duration of the hindlimb (P = 0.81) and the duty factor of the forelimb did not change (P = 0.52). The post hoc analysis revealed that the stride duration and swing duration at an 8% grade were significantly shorter compared to those at other grades (Table 1). In addition, the duty factor of the hindlimb at an 8% grade was significantly higher than that at a 0% grade (P = 0.02). Regarding the forelimb stance duration, the value at an 8% grade was significantly lower than that at a −4% grade (P = 0.04).

**Table 1:** Stride parameters at each grade for forelimbs and hindlimbs.

Parameter	Grade			
	−4%	0%	4%	8%
Stride duration (ms)	505 ± 20.6^a^	502 ± 15.4^a^	497 ± 16.7^a^	487 ± 15.8^b^
Forelimb stance duration (ms)	129 ± 8.4^a^	126 ± 4.7^a,b^	123 ± 5.2^a,b^	123 ± 5.6^b^
Forelimb swing duration (ms)	374 ± 18.7^a^	373 ± 13.9^a^	371 ± 15.0^a^	360 ± 10.0^b^
Hindlimb stance duration (ms)	137 ± 8.4	136 ± 7.6	137 ± 7. 5	135 ± 8.5
Hindlimb swing duration (ms)	369 ± 17.4^a^	366 ± 12.8^a,b^	361 ± 12.7^b^	350 ± 12.7^c^
Forelimb duty factor (%)	25.6 ± 1.4	25.2 ± 1.0	25.0 ± 1.1	25.4 ± 1.0
Hindlimb duty factor (%)	27.0 ± 1.4^a^	27.1 ± 1.37^a^	27.5 ± 1.1^a,b^	27.8 ± 1.4^b^

Data are shown as mean ± standard deviation. Different letters in each row indicate significant differences between each condition.

**Fig 1:**
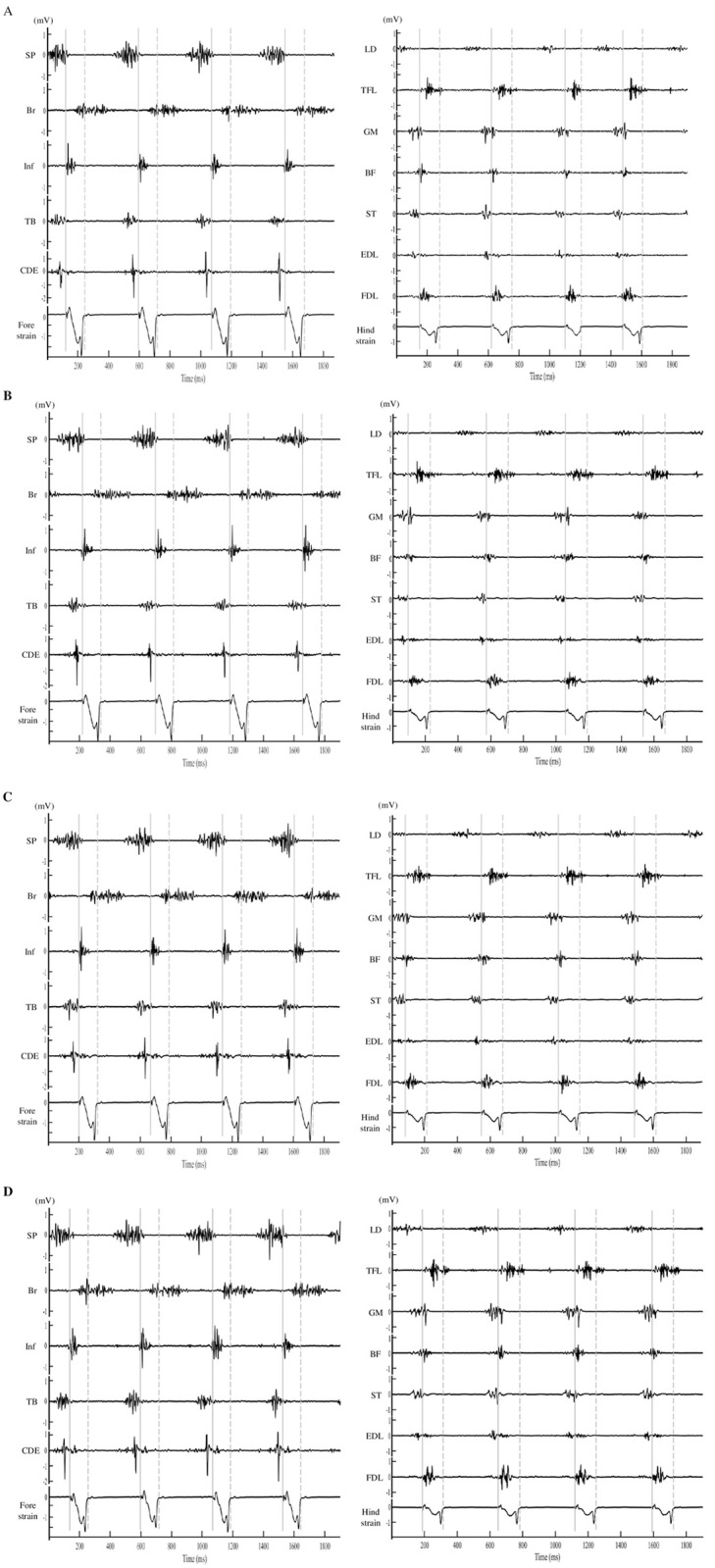
Representative electromyographic activity over 4 consecutive strides in each muscle and strain gauge signals at each grade ((A) −4%, (B) 0%, (C) 4%, (D) 8%) from the same horse. SP; *Musculus splenius;* Br; *M*. *brachiocephalicus*; Inf, *M*. *infraspinatus*; TB, long head of *M*. *triceps brachii*; CDE; *M*. *common digitorum extensor;* LD; *M*. *longissimus dorsi;* TFL; *M*. *tensor fasciae latae;* GM; *M*. *gluteus medius*, BF; *M*. *biceps femoris;* ST; *M*. *semitendinosus*, EDL; *M*. *extensor digitorum longus;* FDL; *M*. *flexor digitorum lateralis*. The gray vertical line, thick and dashed, indicates hoof-on and hoof-off event, respectively. Note that signals were filtered using a band-pass filter (including a Bessel filter ranging 30–500 Hz for EMG) and a low-pass filter set at 250 Hz for the strain gauges.

Fig 1 shows a representative of sEMG waveform for each muscle alongside the strain gauge signal under each condition.

**Fig 2:**
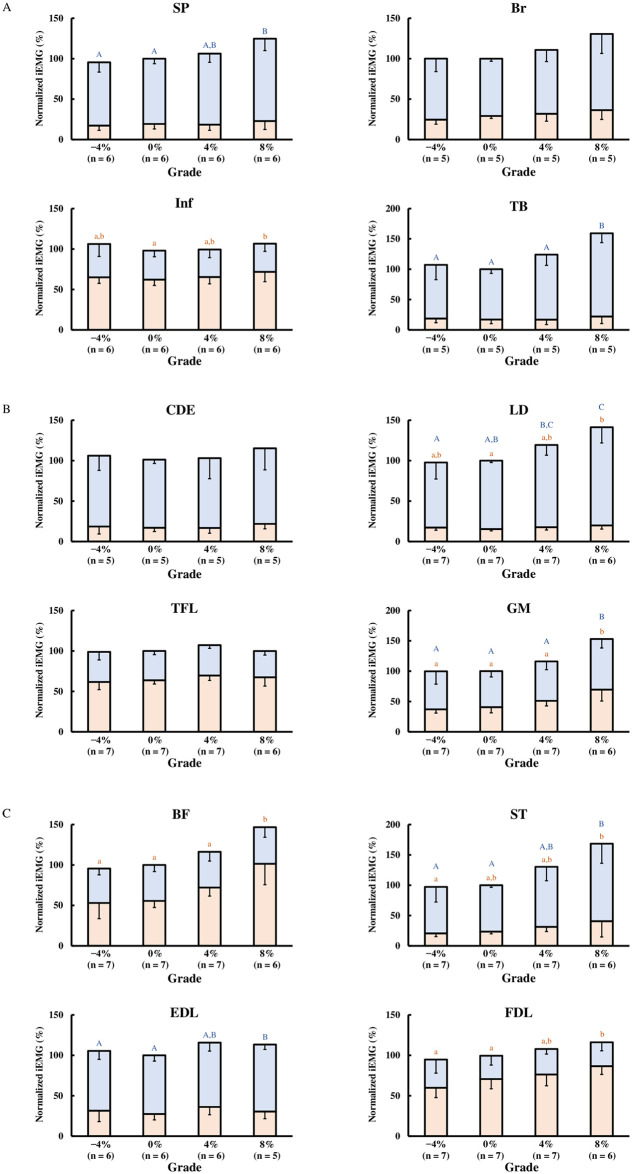
Mean normalized integrated electromyography (iEMG) values during stance (orange color) and swing phase (blue color) on each grade. iEMG values of each muscle during stance phase and swing phase on each grade were normalized to mean iEMG values over stride duration on a 0% grade for each horse. The average normalized iEMG values across horses for each phase are presented with standard deviations. (A) SP; *Musculus splenius;* Br; *M*. *brachiocephalicus*; Inf, *M*. *infraspinatus*; TB, long head of *M*. *triceps brachii*. (B) CDE; *M*. *common digitorum extensor;* LD; *M*. *longissimus dorsi;* TFL; *M*. *tensor fasciae latae;* GM; *M*. *gluteus medius*. (C) BF; *M*. *biceps femoris;* ST; *M*. *semitendinosus*, EDL; *M*. *extensor digitorum longus;* FDL; *M*. *flexor digitorum lateralis*. The use of different letters (uppercase blue-colored letters for the swing phase and lowercase orange-colored letters for the stance phase) indicates significant changes between grades (*P* < 0.05), as determined by the generalized mixed model analysis, followed by Tukey’s post hoc test for pairwise comparisons.

The normalized iEMG value during the stance phase was significantly affected by a grade for the Inf (P = 0.02), LD (P = 0.004), GM (P < 0.001), BF (P < 0.001), ST (P = 0.02), and FDL (P = 0.001) (Fig 2). The post hoc test revealed that the normalized iEMG value on an 8% grade was significantly higher than that on a 0% grade (Inf; +9%, LD; +4%, GM; +29%, BF; +47%, FDL; +16%), while no significant difference was found in ST between 0% and 8% grade (P = 0.06). The normalized iEMG value during the swing phase was significantly affected by a grade in SP (P = 0.02), TB (P < 0.001), LD (P < 0.001), GM (P < 0.001), ST (P = 0.002), and EDL (P = 0.009) (Fig 2). According to the post hoc test, significantly higher normalized iEMG values were observed on an 8% grade compared to a 0% grade in these muscles (SP; +21%, TB; +54%, LD; +37%, GM; +24%, ST; +51%, EDL; +10%). No muscles showed significant changes in normalized iEMG values on −4% grade and 4% grade compared to the 0% grade.

**Fig 3:**
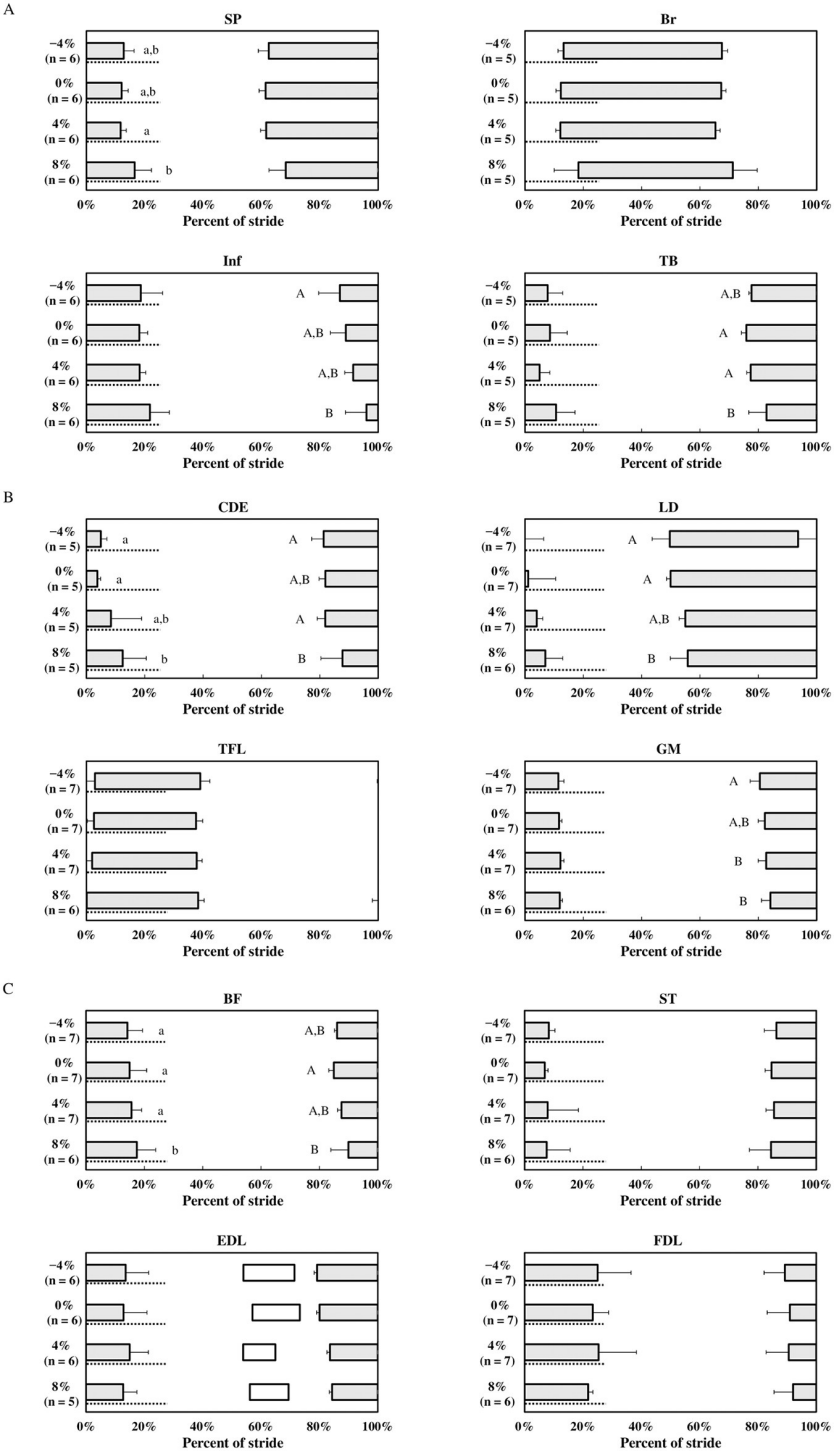
Graphs showing periods of electrical activity of muscles at each grade across a normalized stride cycle (0%–100% hoof strike to subsequent hoof strike). (A) SP; *Musculus splenius;* Br; *M*. *brachiocephalicus*; Inf, *M*. *infraspinatus*; TB, long head of *M*. *triceps brachii*. (B) CDE; *M*. *common digitorum extensor;* LD; *M*. *longissimus dorsi;* TFL; *M*. *tensor fasciae latae;* GM; *M*. *gluteus medius*. (C) BF; *M*. *biceps femoris;* ST; *M*. *semitendinosus*, EDL; *M*. *extensor digitorum longus;* FDL; *M*. *flexor digitorum lateralis*. Average relative periods of electrical activity on each grade are shown. Error bars denote standard deviation. Out of seven horses, four showed additional muscle activation in the EDL, as shown using white boxes. For this additional activity, standard deviations were omitted for clarity. The gray dashed horizontal line shows the stance phase, and the specific values for each limb at each speed, related to the duty factor, are described in Table 1. Different lowercase letters indicate significant changes (P < 0.05) in muscle offset, whereas uppercase letters denote significant changes in muscle onset, as determined by generalized mixed model analysis followed by Tukey’s post hoc test.

Fig 3 shows the onset and offset timing of each muscle across the gait cycle, expressed as a percentage of the total cycle time. Except for Br, LD, and TFL, all muscles are activated before hoof contact and deactivated at some point during the stance phase, regardless of grades. The Br is activated midway through the stance phase and deactivated midway through the swing phase. The LD is activated in the early to middle swing phase and deactivated in the late swing phase or early stance phase. The TFL is activated early in the stance phase and deactivated early in the swing phase. In the EDL, four horses showed an additional phase of muscle activation during the middle of the swing phase.

Tables 2 and 3 show the descriptive statistics, including mean ± standard deviation, for muscle activation timing. The effect of graded surface on the timing of muscle onset was significant in several muscles: Br (P = 0.04), Inf (P = 0.02), TB (P = 0.01), CDE (P = 0.02), LD (P < 0.001), GM (P = 0.004), and BF (P = 0.02). However, post hoc test did not reveal any significant changes between each grade in Br. Notably, muscle onset was delayed at an 8% grade compared to a 0% grade for TB (P = 0.01), LD (P < 0.001), and BF (P = 0.01) by Tukey’s multiple comparisons. In CDE and GM, no significant changes were observed between 0% and 8% grade, while muscle onset on −4% was significantly earlier than 8% (P = 0.03 in CDE and P = 0.003 in GM). No significant changes in muscle onset timing were observed at −4% and 4% grade when compared to a 0% grade.

**Table 2:** Mean ± standard deviation for muscle onset, offset, and activity duration of forelimb muscles at each grade.

Muscles	Variable	Grade				p-value
		−4%	0%	4%	8%	
*M*. *splenius*	Onset timing (% leading forelimb stride)	67.9 ± 3.0	67.3 ± 3.0	67.6 ± 3.2	71.7 ± 5.3	0.07
	Offset timing (% leading forelimb stride)	10.9 ± 3.1^a,b^	10.2 ± 1.9^a,b^	9.9 ± 1.6^a^	14.8 ± 5.1^b^	0.04
	Activity duration (% leading forelimb stride)	43.0 ± 3.4	43.0 ± 3.3	42.3 ± 2.8	43.1 ± 2.4	0.83
*M*. *brachiocephalicus*	Onset timing (% leading forelimb stride)	13.3 ± 1.9	12.3 ± 1.7	12.1 ± 1.6	18.4 ± 8.4	0.04
	Offset timing (% leading forelimb stride)	67.5 ± 3.3	67.3 ± 3.0	65.3 ± 3.5	71.3 ± 9.4	0.19
	Activity duration (% leading forelimb stride)	54.3 ± 2.5	55.0 ± 4.3	53.2 ± 4.8	52.9 ± 1.1	0.53
*M*. *infraspinatus*	Onset timing (% leading forelimb stride)	88.0 ± 6.7^A^	89.9 ± 4.8^A,B^	92.2 ± 2.7^A,B^	96.2 ± 6.9^B^	0.02
	Offset timing (% leading forelimb stride)	17.1 ± 2.1	16.5 ± 2.6	16.6 ± 1.9	20.7 ± 6.4	0.10
	Activity duration (% leading forelimb stride)	29.1 ±8.6	26.6 ± 6.1	24.4 ± 3.5	24.6 ± 3.5	0.14
long head of *M*. *triceps brachii*	Onset timing (% leading forelimb stride)	77.7 ± 0.9^A,B^	75.9 ± 1.7^A^	77.4 ± 1.3^A^	82.8 ± 6.1^B^	0.01
	Offset timing (% leading forelimb stride)	7. 8± 5.2	8.6 ± 5.9	5.0 ± 3.5	10.7 ± 6.5	0.07
	Activity duration (% leading forelimb stride)	30.1 ± 4.8	32.7 ± 7.0	27.7 ± 3.5*	27.9 ± 3.6*	0.005
*M*. *common digital extensor*	Onset timing (% leading forelimb stride)	81.3 ± 4.1^A^	81.9 ± 2.2^A,B^	81.9 ± 2.8^A^	87.8 ± 7.4^B^	0.02
	Offset timing (% leading forelimb stride)	4.9 ± 2.1^a^	3.7 ± 1.1^a^	8.4 ± 10.5^a,b^	12.4 ± 8.1^b^	0.01
	Activity duration (% leading forelimb stride)	23.6 ± 3.5	21.8 ± 1.9	26.5 ± 10.3	24.7 ± 6.4	0.38

The effect of grade on each variable is shown as the p-value. Within each row, significant changes (P < 0.05) detected by Tukey’s multiple comparisons of least-squared means are denoted by superscripts. It is noteworthy that Tukey’s comparison test did not detect significant differences between different conditions for the *M*. *brachiocephalicus* muscle.

* for *M*. *triceps brachi*i of activity duration means significant difference compared to a 0% grade.

**Table 3:** Mean ± standard deviation for muscle onset, offset, and activity duration of hindlimb muscles at each grade.

Muscles	Variable	Grade				p-value
		−4%	0%	4%	8%	
*M*. *longissimus dorsi*	Onset timing (% leading hindlimb stride)	49.7 ± 6.1^A^	49.4 ± 6.4^A^	53.3 ± 6.7^A,B^	55.8 ± 3.5^B^	< 0.001
	Offset timing (% leading hindlimb stride)	93.6 ± 12.8	1.6 ± 7.1	3.9 ± 4.8	7.0 ± 1.2	0.42
	Activity duration (% leading hindlimb stride)	44.0 ± 10.5	52.3 ± 10.9	50.6 ± 10.1	51.2 ± 3.8	0.08
*M*. *tensor fasciae latae*	Onset timing (% leading hindlimb stride)	2.9 ± 3.2	2.6 ± 2.3	2.0 ± 1.9	0.1 ± 2.0	0.36
	Offset timing (% leading hindlimb stride)	39.0 ± 3.1	37.6 ± 3.0	37.8 ± 3.1	38.3 ± 3.5	0.70
	Activity duration (% leading hindlimb stride)	36.1 ± 5.0	35.0 ± 4.1	35.8 ± 4.7	38.2 ± 4.8	0.36
*M*. *gluteus medius*	Onset timing (% leading hindlimb stride)	80.5 ± 3.4^A^	82.2 ± 2.3^A,B^	82.7 ± 2.7^B^	84.2 ± 3.1^B^	0.004
	Offset timing (% leading hindlimb stride)	11.4 ± 0.9	11.7 ± 1.2	12.2 ± 1.0	11.9 ± 2.0	0.45
	Activity duration (% leading hindlimb stride)	30.9 ± 3.8	29.4 ± 2.7	29.5 ± 3.0	27.8 ± 4.4	0.07
*M*. *biceps femoris*	Onset timing (% leading hindlimb stride)	86.1 ± 3.6^A,B^	85. 0± 1.3^A^	87.6 ± 3.2^A,B^	90.0 ± 3.6^B^	0.02
	Offset timing (% leading hindlimb stride)	14.2 ± 1.5^a^	14.9 ± 1.7^a^	15.6 ± 1.5^a^	17.4 ± 2.7^b^	< 0.001
	Activity duration (% leading hindlimb stride)	28.1 ± 2.4	29.9 ± 2.1	28.0 ± 2.1	27.5 ± 1.9	0.17
*M*. *semitendinosus*	Onset timing (% leading hindlimb stride)	86.3 ± 3.7	84.6 ± 2.0	85.5 ± 1.7	84.5 ± 1.3	0.37
	Offset timing (% leading hindlimb stride)	8.3 ± 4.3	6.9 ± 1.5	7.9 ± 1.5	7.6 ± 2.0	0.63
	Activity duration (% leading hindlimb stride)	22.0 ± 2.6	22.3 ± 1.9	22.4 ± 2.0	23.1 ± 1.5	0.33
*M*. *extensor digitorum longus* (main discharge)	Onset timing (% leading hindlimb stride)	79.2 ± 6.3	80.1 ± 7.3	83.6 ± 6.8	84.4 ± 3.2	0.08
	Offset timing (% leading hindlimb stride)	13.6 ± 7.9	12.8 ± 8.1	14.9 ± 6.5	12.7 ± 4.7	0.35
	Activity duration (% leading hindlimb stride)	34.4 ± 7.8	32.8 ± 9.4	31.3 ± 5.8	28.4 ± 4.8	0.45
*M*. *extensor digitorum longus* (Additional discharge)	Onset timing (% leading hindlimb stride)	53.9 ± 1.6	57.1 ± 6.0	53.9	56.2 ± 3.3	N.A.
	Offset timing (% leading hindlimb stride)	71.4 ± 7.1	73.3 ± 1.5	64.9	69.4 ± 6.2	N.A.
	Activity duration (% leading hindlimb stride)	17.5 ± 8.2	16.2 ± 5.1	11.1	13.3 ± 2.9	N.A.
*M*. *flexor digital lateralis*	Onset timing (% leading hindlimb stride)	89.5 ± 6.9	91.4 ± 7.4	90.8 ± 7.5	92.7 ± 6.0	0.23
	Offset timing (% leading hindlimb stride)	24.4 ± 11.1	22.2 ± 5.2	24.7 ± 12.6	20.1 ± 1.5	0.90
	Activity duration (% leading hindlimb stride)	34.9 ± 14.3	30.9 ± 11.3	33.8 ± 18.4	27.4 ± 6.9	0.65

The effect of grade on each variable is shown as the p-value. Within each row, significant changes (P < 0.05) detected by Tukey’s multiple comparisons of least-squared means are denoted by superscripts.

The analysis of offset timing revealed that the effect of graded surfaces was significant for several muscles: SP (P = 0.04), CDE (P = 0.01), and BF (P < 0.001). Post hoc test revealed that cantering on an 8% grade significantly delayed the muscle offset in CDE (P = 0.009) and BF (P = 0.003), compared to a 0% grade. In SP, no significant change was observed between 0% and 8% grade, while muscle offset on an 8% was significantly delayed when compared to a 4% grade (P = 0.04). No significant changes were observed in muscle offset timing at −4% and 4% grade when compared to a 0% grade.

The relative duration of activity for the long head of TB was significantly influenced by the grade (P = 0.005). There were no significant differences in other muscles. Post hoc test revealed that cantering on 4% and 8% grade significantly shortened the relative activity duration of TB compared to a 0% grade (P = 0.008 and 0.01, respectively).

## Discussion

The present study examined the effects of graded surfaces on stride parameters and muscle activity during canter in Thoroughbred horses. Compared to a 0% grade, stride duration significantly decreased on an 8% grade due to a decrease in swing duration, accompanied by an increase in the duty factor of the hindlimb. On this steeper grade, muscle activity mainly in the hindlimb increased during the stance phase, and some muscle activity in both the forelimb and hindlimb increased during the swing phase. Furthermore, no significant changes in stride parameters and muscle activity were observed at either −4% or 4% incline when compared to a 0% incline.

When horses gallop on a 10%–15% grade, the estimated mechanical work, which is the sum of kinetic energy and potential energy, has been observed to be significantly greater than that on a level surface at a given speed [10]. This increase was primarily explained by the additional work required to move the animal up a slope (i.e., potential energy), especially during the stance phase of the leading hindlimb, which experienced a larger peak vertical ground reaction force compared to a level surface [10, 19]. Among the hindlimb surface muscles we investigated in the current study, GM, BF, and FDL could play an essential role in acquiring potential energy when horses canter on a steep inclined surface, as their activation during stance phase on an 8% grade significantly increased. Furthermore, the activity in Inf during the stance phase increased on an 8% grade compared to a 0% grade, suggesting that some positive work necessary for gaining potential energy can be performed by forelimb muscles. These results indicate that the shoulder, hip and hindlimb fetlock joint may play a significant role in elevating equine center of mass during uphill canter.

Our study uncovered an interesting aspect regarding the different muscle activity patterns of hip joint extensor muscles when horses galloped on an 8% grade. Compared to a 0% grade, normalized iEMG values on an 8% grade during the swing phase significantly increased in GM and ST muscles. These muscles may play an important role in decelerating the limb and redirecting it from protraction to retraction in the late swing phase. This function is similar to the role of the long head of TB, where a shorter swing duration necessitates faster limb protraction [35, 36]. This indicates that not only passive properties, such as the biceps brachii tendon unit’s catapult mechanism, but also an increase in muscle activity, could facilitate faster limb movements on an uphill grade [10, 37]. Although the functions of the BF muscle, which include hip extension during the stance phase, stifle flexion, and hock extension during the swing phase, are similar to those of the ST muscle [25, 38], muscle activity in BF during the swing phase did not significantly change. This indicates that the contribution of the BF muscle to faster limb movement may be less than that of the ST muscle. Additionally, the role of BF for extending the tarsal joint, which occurs from the middle to the late stance phase [39], may be more significant than that of the ST muscle. This is when the activity of the BF muscle was observed, whereas the activity of the ST muscle had already finished.

Hodson–Tole demonstrated that when horses canter at 7.2 m/s on an 8% grade, the EMG intensity in the long head of TB and Br significantly increased compared to level running, presumably due to increased forelimb protraction and retraction angles [18, 20]. Our results in TB align with these findings. We observed that the normalized iEMG values during the swing phase on an 8% grade significantly increased compared to a 0% grade, indicating that TB may play an important role in decelerating the forelimb [36]. Contrary to Hodson–Tole’s findings [20], the activity of Br was not affected by grade in our study. While Hodson–Tole measured trailing limb side muscle activity [20], we measured the leading limb side. This discrepancy suggests that the response to graded surface might differ between the leading and trailing forelimbs.

The observed significant increase in normalized iEMG value in SP during the swing phase and in LD during both stance and swing phases on an 8% grade indicates that steep uphill cantering requires horses to increase activity of stabilizing their trunk more than that required for level cantering, a finding that aligns with observations during trotting [14]. The SP was activated from the middle swing phase to the early stance phase of the ipsilateral limb, which is consistent with a recent report [40]. This suggests that SP can reverse the neck segment rotation and may also contribute to the vertical lifting of the center of mass during the upcoming suspension phase [41]. According to Parsons et al., galloping on a 10%–15% incline increased the trunk pitch range and maximum pitch angular velocity compared to galloping on a 0%–2% incline [10], suggesting greater trunk rotation on inclines during a gallop. This implies an increased need for SP muscle activity to counteract this rotation. When horses canter and gallop, LD is typically active once per stride during the suspension phase and the trailing hindlimb stance phase [42]. The function is to prepare for the landing of the hindlimbs and to extend the trunk before the forelimbs land, resulting in stabilizing the thoracolumbar spine [42], which is consistent with our results. The significant increase in the normalized iEMG values of LD mainly during the swing phase suggests more sagittal plane movement (i.e., extension and flexion) in the back after the ipsilateral hindlimb hoof-off and increased stiffness of the equine back preparing the horse for landing [43, 44]. Furthermore, the increase in trunk muscle activity helps to create a rigid platform, enabling the limbs to swing faster [45].

No direct relationship between muscle activation and grade was observed in TFL and CDE. The primary function of these muscles is believed to be joint stabilization [27, 46], which may be less important for generating potential energy on grades. Although the main function of EDL is to flex the tarsal joint and extend the digit, which shares similar roles with the CDE [27, 47], its activity during the swing phase significantly increased on steep grades. This suggests that the contribution of the EDL to flexing the tarsal joint is substantial, likely due to a greater range of motion in the tarsal joint, as indicated by results obtained during trotting [48]. To fully understand these different responses among different muscles, further studies incorporating kinematic analysis or musculoskeletal modeling to estimate muscle forces are warranted.

On an 8% grade, muscle onset was significantly delayed in TB, LD, and BF when compared to a 0% grade. In these muscles, relative sEMG activity duration during swing phase was shorter on an 8% grade than a 0% grade. Despite shorter activity duration, normalized iEMG values during swing phase in TB and LD significantly increased on an 8% grade. In addition, normalized iEMG values during swing phase in SP, GM, ST, and EDL on an 8% grade were significantly larger than a 0% grade, although muscle onset timing did not differ in these muscles between 0% and 8% grade. This suggests that increase of iEMG values during swing phase observed in the current study would be explained not by increased muscle activation time but by increased muscle fiber recruitment. Additionally, muscle offset was significantly delayed on an 8% grade in CDE and BF, indicating that relative sEMG activity duration during stance phase would be longer when compared to a 0% grade. Although statistically significant differences were detected, it is important to note that changes in the timing of muscle activation relative to the limb cycle are generally small compared to the increase in EMG intensity [24, 49–51]. Daley et al. interpreted this as reflecting an increase in muscle recruitment to meet the greater work requirements of incline locomotion [51]. This interpretation appears to apply to our study as well, because in Inf, LD, GM, and FDL, which showed significant increase of iEMG values during stance phase on an 8% grade compared to a 0% grade, the relative activity duration during stance phase remained consistent.

Our findings revealed that compared to a 0% grade, there were no significant changes in any muscle activity at a 4% or –4% grade. This suggests that slight changes in the grade of the surface may have minimal impact on equine neuromuscular adaptation during canter, which is further supported by the fact that there were no changes in any stride parameters under these conditions. Alternatively, this observation could be attributed to the characteristics of the small and superficial electrodes used in the study. Supporting this second explanation, our previous research indicated that running on a 4% decline reduced mass-specific oxygen consumption by 24%, whereas a 4% grade led to a 49% increase compared to level running [23]. With respect to downhill running, the current study would not be able to draw firm conclusion about neuromuscular response since we did not perform −8% condition, while we measured muscle activity on an 8% grade. This is because some horses were unable to perform steady canter at 10 m/s on an 8% declined treadmill. Indeed, evidence from studies on rats indicates that a steep decline during canter might reduce muscle activity [24]. Further study would be needed to elucidate equine neuromuscular response to downhill running.

Cantering on an 8% grade resulted in a significant increase in muscle activity during both the stance phase (Inf, LD, GM, BF, and FDL) and the swing phase (SP, TB, LD, GM, ST, and EDL) compared to cantering on a 0% grade. These suggest that training on an 8% grade may cause large neuromuscular responses for athletic horses to strengthen these muscles to increase their propulsive force and stride frequency, which may improve athletic performance with less load to forelimbs [19]. However, even if peak estimated ground reaction force in forelimbs during 8–12% canter decreased by 11–12% when compared to 0–2% [19], cantering on an 8% grade may not be appropriate for horses with forelimb muscle injuries in Inf or TB.

Our observations that the stance time remained constant across graded surfaces, coupled with a gradual increase in the duty factor of the hindlimbs and a decrease in swing duration on an 8% grade, which indicates an increased stride frequency, were consistent with previous literature on galloping horses and humans [19, 33]. In addition to the increased muscle activity we observed, swing duration may also be reduced as a result of the surface hindering the limb’s arc during repositioning for the subsequent stance phase, which occurs earlier because of the inclined ground [19]. The consistency in contact time, despite varying grades, may be a strategy to minimize the energetic cost of graded running, as active muscle energy use during running is known to be inversely proportional to contact time [52]. However, these findings appeared to contrast with those reported in trotting horses [15, 17, 53]. Studies have shown that during trotting on a 10% incline, both stance time and stride duration tend to be longer compared to level trotting [15, 17, 53]. To gain power during uphill trotting, horses increase their work per cycle [15], whereas during cantering, they can increase the number of cycles as well as the work per cycle. This suggests that the response to graded surfaces during cantering might be slightly different from trotting in terms of stride parameters, although the muscular recruitment pattern could be similar. The changes in stride parameters in response to graded surfaces appear to depend on the gait or the type of animal involved [7, 24, 33, 54].

The present study has several limitations. First, the relatively small sample size may limit the generalizability of our findings to the broader equine population. Second, our results were derived only from the preferred leading limb side at a set speed. We measured muscle activity in the leading limb side because the leading hindlimb may be more important than the trailing hindlimb in generating potential energy, especially since an increase in potential energy on 8%–12% inclines compared to 0%–2% inclines was observed during the leading hindlimb stance phase [10]. However, considering that canter and gallop are asymmetric gaits, wherein muscle activity and kinematics significantly differ between leading and trailing limbs [36, 40, 55, 56], our approach may overlook important aspects of equine locomotion. Further, some studies have shown that an interaction between graded surfaces and speed affected muscle activity and range of motion in human running [33, 57]. For a comprehensive understanding of the effects of graded surfaces during canter in horses, future research should investigate the interactions between grade, speed, and whether a limb is leading or trailing. Third, we have to recognize we could not rule out all motion artifacts including displacement of the motor point under electrodes, despite applying recommended filtering cutoff frequency [58]. Also, the signal processing method we applied for detecting muscle onset and offset, which was originally developed for BF and TB [34], might have overlooked some muscle activations, especially when amplitudes were small and the peak values were considerably high. Finally, it is unclear whether our results can be applied to regular overground training, because treadmill exercise may demand more limb retraction than overground movement during trotting [59], which might induce early muscle deactivation in TB [47]. A comparative analysis of muscle activations between treadmill and overground cantering is warranted to address this gap.

## Conclusions

In conclusion, our study successfully characterized the neuromuscular responses of horses to graded surfaces during canter. When compared to a 0% grade, the effect of 4% grades (both downhill and uphill) on muscle activity was not significant. In contrast, during an 8% grade canter, horses need to expedite limb movement due to a shorter swing duration by increasing muscle activity of SP, TB, LD, GM, ST, and EDL. Additionally, in order to gain potential energy, muscle activity in Inf, LD, GM, BF, and FDL significantly increased during stance phase. Therefore, cantering on an 8% grade may cause large neuromuscular responses for athletic horses to strengthen muscles to increase propulsive force and stride frequency, which may improve athletic performance.

## Supporting information

S1 Appendix The electrode locations for each muscle. (DOCX)

S1 Table Raw data as used to build graphs and perform statistical analysis. (XLSX)
