# Supplementary material for: Changes in muscle activation with graded surfaces during canter in Thoroughbred horses on a treadmill
Source: PLoS One. 2024 Jun 14;19(6):e0305622. doi: 10.1371/journal.pone.0305622 (PMC11178216; doi:10.1371/journal.pone.0305622)
Supplement: S1 Appendix — (DOCX) [file pone.0305622.s001.docx]

Electrode locations for each muscle

*Musculus splenius*: At the level of the axis

*M. brachiocephalicus*: At the midpoint of an imaginary line drawn from the atlas to the edge of the shoulder

*M. infraspinatus*: Just caudal to the spine of the scapula

Long head of *M. triceps brachii*: At the midpoint of an imaginary line drawn from the proximal point of the scapular spine to the olecranon

*M. common digitorum extensor*: Just cranial to the lateral tuberosity of the radius

*M. longissimus dorsi*: At the level of the 16th thoracic vertebra

*M. tensor fasciae latae*: Just under the tuber coxae, within the muscular part of this muscle, which is less likely to be affected by skin movement

*M. gluteus medius*: On an imaginary line drawn from the tuber coxae to the root of the tail, at one-third of the distance from the tuber coxae

*M. biceps femoris*: On an imaginary line drawn from the tuber coxae to the root of the tail, at its upper third portion

*M. semitendinosus*: At the level of the tuber ischii

*M. extensor digitorum longus*: Just under the tibial tuberosity

*M. flexor digitorum lateralis*: Just cranial to the common calcaneal tendon
